# Sir George Newman on the Ministry of Health's Position

**Published:** 1920-10-23

**Authors:** 


### AN INSTRUMENT IN PREVENTIVE MEDICINE.

### Sir George Newman on the Ministry of Health's Position.

On Wednesday evening, the 13th inst., the first meeting of the Hunterian Society in the present session was held at Sion College, Embankment, under the presidency of Dr. A. C. Jordan, who, in introducing Sir George Newman, referred with satisfaction to the fact that the Society had now, in its second century of life, obtained a permanent resting-place at the College.

Sir George Newman explained that John Hunter left three legacies to succeeding generations of practitioners, which formed a kind of basis for the new conception of preventive medicine, a subject not new in itself, but only so in its outlook. Hunter was a student of the evolution of function; secondly, he insisted on the importance of comparative pathology; and he taught that healing was dependent on the recuperative and defensive powers of the body.

### The Medical Structure of the Ministry.

The lecturer entered in considerable detail into the medical structure of the Ministry of Health. During eighty years, he said, there have been medical departments attached to different central offices of government, and the chief of these have now been combined in a Ministry devoted to health itself. The purpose of that Ministry is to promote the health of the people, to take all such steps as may be desirable to secure the preparation, effective carrying-out, and the co-ordination of measures conducive to the health of the community, including the following seven functions:

1. The prevention and cure of disease.
2. The avoidance of fraudulent remedies.
3. Treatment of physical and mental defects.
4. The treatment and care of the blind.
5. The initiation and direction of medical research.
6. The collection and publication of information and statistics with regard to public medicine.
7. The training of persons for the public health service.

Medical education has not been omitted, but at the time the Bill was framed medical education had been for ten years under the Board of Education. It has now been transferred to the University Grants Committee of the Treasury, which can deal with Scotland and Ireland, as well as England and Wales.

An arrangement has been made by which the Ministry's medical work is separated into six main divisions, with a medical person in charge, a staff of 105 medical people carrying out their directions. There is: (1) A general health and epidemiology division, with a laboratory for research; (2) a maternity and child-welfare department, staffed entirely by medical women, nurses, and midwives; (3) a department for tubercle and venereal diseases; (4) for the control of food supply; (5) for general medical practice; (6) for sanitary administration. A school medical service has now been added, and the Registrar-General's department and the Lunacy Board of Control have been transferred. The Medical Consultative Committee, under the chairmanship of Lord Dawson, is the body of which the Minister can ask advice when he wishes to go outside the permanent staff, and Sir George had a word of welcome for this new body.

For the first time, also, medical referees have now been appointed, who will represent the medical practitioners in the country, and will be located in various parts of the kingdom.

The medical staff of the Ministry are working together to get information, and it is duly studied and charted. The system has also been extended to embrace the whole world, and telegrams are received from British Consuls regularly, giving important medical material. Regular reports are received in a similar manner from all the local authorities of the kingdom, in which are now 1,800 sanitary authorities, 318 local education authorities, 650 Poor-law Unions, 245 Pension Committees, and 142 Insurance Committees. That five different agencies should be at work in the same districts does not tend to economical working, and steps are to be taken as soon as possible to remedy this.

### A Point of Emphasis.

Sir George emphasised the fact that the function of the Ministry is not to solve the problems of local self-government, but to solve those arising from central medical administration having been carried on by many different departments of State. The Ministry is building for the future. The Ministry can only work through the local authorities: it cannot "deliver the goods"; it comes between the Legislature and the local authorities, and these beneficent schemes can only be effectual by the will of the people.

In elaborating his theme the lecturer gave some striking instances of the material at hand, and explained how it was being dealt with. He said the three guiding principles are to strengthen the resources of the body, to avoid infection, and to educate the people. Efforts are being made to render the sanitary cordon round these islands complete, especially in reference to food and aliens, and to create an International Health Office, to be attached to the League of Nations.

With regard to maternity work, 3,000 mothers die yearly in childbirth, the number of cases of abortion, miscarriage, still-birth, and ophthalmia neonatorum are much too high, and there is still a heavy loss of infants under one year of age. The solution of this problem seems to lie along the path of care and inspection of mothers by practitioners in a systematic way, and the provision of a greatly increased maternity accommodation, which at present is deplorably short.

In regard to mental disease, he said there were in this

**An Instrument in Preventive Medicine**—(*continued*).

country's various asylums 116,000 insane persons, and another 150,000 feeble-minded (35,000 of the latter being children). Fully 10 per cent. of the school children are said to be inherently "dull and backward," and many people have incipient mental aberration, which, unless checked, may develop into serious mind disorder. He thought Clouston's advice should be followed—namely, that remedial action should take place at a stage somewhere between normality and actual certifiable insanity.

The lecture concluded with details of the improvements recently effected in regard to the medical insurance service, and at the close Sir George was accorded a warm vote of thanks.

